# Mediterranean Diet Adherence and Sleep Quality in Spanish Adults: Divergent Associations Between Subjective and Objective Measures

**DOI:** 10.3390/nu18203305

**Published:** 2026-10-09

**Authors:** Rosario Alonso-Domínguez, Jesús González-Sánchez, César Jiménez-Vaquero, Irene Ailing García-Yu, Natalia Sánchez-Aguadero, Andrea Crespo-Sedano, José Ignacio Recio-Rodríguez

**Affiliations:** 1 Departamento de Enfermería, Facultad de Enfermería y Fisioterapia, Instituto de Investigación Biomédica de Salamanca (IBSAL), Universidad de Salamanca, Campus Miguel de Unamuno s/n, 37007 Salamanca, Spain; ralonsod@usal.es (R.A.-D.); jesusgonzsan@usal.es (J.G.-S.); cesar.jimenez@usal.es (C.J.-V.); ailatan@usal.es (N.S.-A.); donrecio@usal.es (J.I.R.-R.); 2 Departamento de Ciencias Biomédicas y del Diagnóstico, Facultad de Medicina, Instituto de Investigación Biomédica de Salamanca (IBSAL), Universidad de Salamanca, Campus Miguel de Unamuno s/n, 37007 Salamanca, Spain; 3 Complejo Asistencial Universitario de Palencia, Avd. Donantes de Sangre s/n, 34005 Palencia, Spain; acrespos@saludcastillayleon.es

**Keywords:** sleep quality, sleep efficiency, Mediterranean Diet, actigraphy

## Abstract

**Objective:** We aimed to examine the association between adherence to the Mediterranean diet (MD) and sleep quality assessed using both subjective and objective methods in a general adult population. **Design:** This is a cross-sectional study. Adherence to the MD was evaluated using the Mediterranean Diet Adherence Screener, while sleep quality was assessed subjectively using the Pittsburgh Sleep Quality Index and objectively using wrist actigraphy over five consecutive days. Multivariable linear and logistic regression models were applied, adjusting for key sociodemographic and clinical confounders. **Settings:** We investigated a general adult population from Spain with a total of 500 Spanish adults aged 25–65 years. **Results:** Higher MEDAS scores were significantly associated with better subjective sleep quality. Each one-point increase in MD adherence was associated with lower PSQI scores, and participants with higher adherence had lower odds of poor sleep quality, although this association did not reach statistical significance. Cluster analysis identified a dietary pattern characterized by higher consumption of fruits, vegetables, fish and olive oil that was associated with better perceived sleep quality. No significant associations were observed between MD adherence and objective sleep parameters, including sleep efficiency, sleep fragmentation or wake after sleep onset. **Conclusions:** Greater adherence to the MD was associated with better perceived sleep quality but not with objectively measured sleep parameters. These findings suggest that dietary patterns may influence sleep perception rather than objective sleep characteristics and highlight the potential role of the MD as a modifiable factor related to sleep-related well-being.

## 1. Introduction

### 1.1. Sleep as a Determinant of Health

Sleep is a biological process essential for physiological, cognitive, and emotional homeostasis [1]. It is a complex phenomenon regulated by interactions between circadian and homeostatic mechanisms that determine sleep structure and duration [2]. Sleep quality not only reflects the efficiency of nighttime rest but also influences multiple biological systems, including immune function, metabolic regulation, neural plasticity, and cardiovascular health [3]. Chronic sleep disruption has been associated with an increased risk of obesity, type 2 diabetes, hypertension [4], cardiovascular disease [5], cognitive decline [6], and mood disorders [7], constituting a significant risk factor for morbidity and mortality in the adult population.

From a neurobiological perspective, sleep regulation involves the interaction of multiple neurotransmitters and hormones, including serotonin, dopamine, GABA, and melatonin [8]. Disruption of these systems can lead to alterations in sleep architecture and the subjective perception of insufficient rest, even when objective measures do not show significant changes [9]. Therefore, understanding the determinants of sleep and the factors that influence both its physiological and perceived quality is a central objective in research on well-being and chronic disease prevention.

### 1.2. Sleep Assessment: Subjective vs. Objective Measures

Sleep assessment can be performed using objective instruments, such as actigraphy or polysomnography. Actigraphy provides information on sleep efficiency, latency, nighttime awakenings, and total sleep duration via motion sensors [10], while polysomnography directly records brain electrical activity, heart rate, respiration, and other physiological parameters throughout the night [11]. Although these techniques are highly accurate, their application in population-based studies is limited due to their high cost, logistical complexity, and the need for specialized equipment [12].

In contrast, subjective measures, such as the Pittsburgh Sleep Quality Index (PSQI), allow for the assessment of an individual’s perception of sleep in terms of duration, efficiency, perceived quality, latency, and daytime sleepiness [13]. These questionnaires capture the personal experience of sleep, a component that is not always reflected in physiological recordings [14]. Current evidence has documented discrepancies between objectively measured and perceived sleep [15], highlighting that subjective sleep perception can be influenced by psychological factors and mood states [16]. Therefore, assessing sleep from a subjective perspective is crucial for understanding how individuals experience and value their rest, as well as how behavioral factors may modify this perception.

### 1.3. The Mediterranean Diet: Nutritional Profile and Health Effects

The Mediterranean diet (MD) is a dietary pattern characterized by high consumption of fruits, vegetables, legumes, whole grains, extra virgin olive oil, nuts, and fish, along with low consumption of red meat, processed meats, and ultra-processed foods [17]. Its nutritional profile includes a high content of monounsaturated and polyunsaturated fatty acids, phenolic antioxidants, dietary fiber, and essential micronutrients that exert anti-inflammatory, antioxidant, and neuromodulatory effects [18].

Numerous epidemiological studies and clinical trials have shown that adherence to the MD is associated with a lower incidence of cardiovascular disease, metabolic syndrome [19], type 2 diabetes [20], and depression [21]. Furthermore, emerging evidence suggests that this dietary pattern may influence sleep quality, potentially through the modulation of neurotransmitters involved in sleep regulation, reduction in oxidative stress, decreased systemic inflammation, and improvements in overall metabolic health [22]. These mechanisms provide a plausible biological basis for the association between the MD and better perceived nighttime rest, particularly regarding subjective sleep reported by individuals.

### 1.4. Evidence on the Relationship Between the Mediterranean Diet and Subjective Sleep

The recent literature indicates that greater adherence to the MD is associated with higher scores on self-reported sleep questionnaires, including the PSQI, as well as a lower prevalence of insomnia, daytime sleepiness, and difficulty falling asleep [23,24]. However, findings based on objective measures, such as actigraphy, are less consistent, suggesting that the benefits of the MD may be more clearly reflected in the subjective perception of sleep quality than in strictly physiological parameters [25]. This highlights the importance of considering both personal sleep experience and physiological measurements in nutritional studies, as the perception of rest can directly influence overall well-being, adherence to healthy behaviors, and mental health.

Despite these findings, most existing studies are cross-sectional, have methodological limitations, and show heterogeneity in both the assessment of adherence to the MD and the characteristics of the study population. To the best of our knowledge, this is the first study to explore the relationship between MD and sleep quality in a large population using both subjective (self-reported questionnaires) and objective (actigraphy) measures.

Therefore, the objective of this study was to analyze the relationship between adherence to the MD and sleep quality, assessed using both self-reported measures and actigraphy, in a random sample of the general Spanish population aged 25 to 65 years, stratified by sex and age.

## 2. Materials and Methods

### 2.1. Study Design

A cross-sectional observational study was conducted in two locations in central Spain: Ávila and Salamanca. The study was carried out between May 2022 and April 2024 and was registered on the ClinicalTrials.gov platform (NCT05324267), as detailed in the previously published protocol [26].

### 2.2. Study Population and Recruitment

The final sample consisted of 500 adults aged 25 to 65 years, residing in Salamanca and Ávila. Participants were selected through stratified random sampling by age and sex, using the health card database of Castilla y León Health Service (SACYL). One hundred individuals were included in each age group (25, 35, 45, 55, and 65 years), with an equal distribution between men and women, and were contacted by telephone. Those who declined to participate were replaced until the planned sample size was reached. The distribution by age group and sex is shown in Figure 1.

#### 2.2.1. Inclusion Criteria

Individuals aged 25, 35, 45, 55, and 65 years at the time of sampling were included. Participants were required to be registered in the Castilla y León Health Service (SACYL) database, reside in Salamanca or Ávila (Spain), provide written informed consent, and not meet any of the predefined exclusion criteria.

#### 2.2.2. Exclusion Criteria

Participants with terminal illnesses, those unable to attend the centers for the required assessments, and pregnant women were excluded.

### 2.3. Study Variables

#### 2.3.1. Sociodemographic Variables

Age, sex, marital status, education level, and employment status were recorded.

#### 2.3.2. Adherence to the Mediterranean Diet

Adherence was assessed using the Mediterranean Diet Adherence Screener (MEDAS) [27], consisting of 14 dichotomous items. The MEDAS has been validated in Spanish population and showed moderate agreement with a validated food-frequency questionnaire (intraclass correlation coefficient = 0.51). Twelve items assess the frequency of consumption of specific foods, and two items address dietary habits characteristic of the MD. One point was awarded for: using olive oil as the primary cooking fat; preferring white meat over red meat; daily consumption of four or more tablespoons of olive oil; two or more servings of vegetables; three or more pieces of fruit; less than one serving of red meat or processed meats; less than one serving of animal fat; and less than one glass of carbonated or sugary beverages. One point was also awarded for weekly consumption of: seven or more glasses of wine; three or more servings of legumes; three or more servings of fish; two or fewer servings of industrial pastries; three or more servings of nuts; and two or more servings of sofrito (a sauce made with tomato, garlic, onion, or leek, sautéed in olive oil). The final score ranged from 0 to 14 points, with a score of ≥9 points considered indicative of adequate adherence to the MD.

#### 2.3.3. Sleep Assessment

##### Actigraphy

Sleep was objectively assessed using an ActiGraph^®^ device (ActiGraph, Pensacola, FL, USA), which is widely recognized for its reliability in sleep research [28]. The device was placed on the participants’ non-dominant wrists during the study visit, and participants were instructed to wear it for the following five consecutive days, including at least one weekend day. Data were subsequently processed using ActiLife 6 software (v6.13.15), applying the Cole–Kripke [29] and Tudor–Locke [30] algorithms, which automatically identify sleep and wake periods. The following sleep parameters, among others, were derived: time in bed (minutes spent in bed, from the time they go to bed until they get up); total sleep time (minutes of actual time a person is asleep); awakenings (number of times individuals wake up during the sleep period); sleep efficiency (percentage of time spent asleep relative to total time in bed); fragmentation index (ratio of the number of 1-min immobility phases to the total number of immobility phases of all durations multiplied by 100); wake after sleep onset (WASO; total duration of wake time occurring after sleep onset); and movement index (number of minutes with one or more movements divided by total time in bed and multiplied by 100).

##### Pittsburgh Sleep Quality Index (PSQI)

The PSQI questionnaire [31], validated in Spanish, was used to assess subjective sleep quality. The Spanish version showed adequate internal consistency (Cronbach’s α = 0.81) [32]. It consists of 19 items grouped into 7 components: subjective sleep quality, latency, duration, efficiency, disturbances, medication use, and daytime dysfunction. Each component is scored from 0 (no difficulty) to 3 (severe difficulty), yielding a total score ranging from 0 to 21 points, with higher scores indicating poorer sleep quality. In general, a score > 5 is considered indicative of poor sleep quality.

#### 2.3.4. Morbidity Variables

The presence of comorbidities was determined using the Charlson Comorbidity Index (CCI) [33], a validated weighted index that considers the number and severity of comorbid conditions to estimate mortality risk. Higher scores indicate a greater comorbidity burden and a higher predicted risk of mortality. In the original validation study, scores were grouped as 0, 1–2, 3–4, and ≥5, showing a stepwise increase in mortality with increasing CCI scores. The total score (0–37) is adjusted by adding one point for each decade of age starting at 50 years.

### 2.4. Sample Size Estimation

The sample size was determined based on the mean PSQI scores reported by Gómez-García et al. in a cohort of Spanish nurses (6.80 ± 3.38) [34]. Using this information, it was estimated that a random sample of 500 participants would allow estimation of the population mean with a 95% confidence level and a precision of ±0.32 units, assuming a standard deviation of 3.38. A 15% dropout rate was also considered.

### 2.5. Statistical Analysis

The statistical analysis was conducted in accordance with the study protocol [26]. The distribution of variables was assessed using the Kolmogorov–Smirnov test.

All quantitative variables were assessed for normality and are expressed as means ± standard deviation, while categorical variables are expressed as counts and percentages.

Multivariable regression models were performed with different sleep quality parameters as dependent variables and the MD adherence score as the independent variable. A logistic regression model was also performed using PSQI divided into two categories [<5 and ≥5 (poor sleep quality)] as the dependent variable and the MEDAS score divided into two categories [<9 and ≥9 (high adherence)] as the independent variable.

A two-stage cluster analysis was performed including the 14 items of the MD adherence questionnaire, which identified two distinct clusters.

Finally, differences in sleep quality parameters between clusters were analyzed using analysis of covariance (ANCOVA).

All statistical models were adjusted for the main determinants of sleep quality, including age, sex, education level, employment status, CCI, body mass index (BMI), caffeine and alcohol consumption, smoking status, and physical activity, as potential confounders.

A significance level of α = 0.05 was set as the threshold for statistical significance for all hypothesis tests. Data were analyzed using IBM SPSS Statistics for Windows, Version 30.0 (IBM Corp., Armonk, NY, USA).

## 3. Results

This study included 500 participants. Table 1 presents their sociodemographic characteristics. Most participants were married or in a stable relationship (55.2%), followed by single individuals (37.0%). In terms of educational attainment, 48.8% had a college degree and 40.6% had completed high school. Regarding employment status, 65.6% were employed, 17.4% were retired, and 11.0% were unemployed. The mean CCI score was 0.41 ± 0.88, indicating a low overall comorbidity burden in the study population.

Table 2 presents data on adherence to the MD. The mean MEDAS score was 8.8 ± 2.2, and 57.2% of participants showed high adherence (score ≥ 9). Most participants reported using olive oil as their primary fat source (95.8%), with 75.8% consuming at least 4 tablespoons per day. Additionally, 84.2% reported moderate consumption of red meat (less than one serving per day), and 83.2% reported low consumption of sugary drinks (less than one per day). In contrast, only 38.2% consumed at least 3 servings of legumes per week, and 38.6% consumed three or more pieces of fruit per day.

Regarding the relationship between adherence to the MD and sleep quality (Table 3), a significant association was observed between the MEDAS score and subjective sleep quality (PSQI) in multivariable regression models adjusted for age, sex, educational level, employment status, CCI, BMI, daily caffeine, alcohol consumption, smoking status and physical activity levels. Each one-point increase in the MEDAS score was associated with a 0.187-point decrease in the PSQI score (β = −0.187; 95% CI: −0.354 to −0.021; *p* = 0.027), indicating better sleep quality. No significant associations were observed with objective sleep parameters.

Furthermore, logistic regression models showed that high adherence to the MD (score ≥ 9) was associated with lower odds of poor subjective sleep quality, although this association did not reach statistical significance (OR = 0.685; 95% CI: 0.454 to 1.034; *p* = 0.072), after adjustment for sex, age, educational level, employment status, CCI, BMI, caffeine and alcohol consumption, smoking status, and physical activity.

Cluster analysis (Figure 2) identified two adherence groups (Cluster 1 = 50.6%; Cluster 2 = 49.4%). Cluster 2 exhibited a healthier profile, characterized by higher consumption of fish (72.1%), fruits (63.2%), vegetables (72.9%), olive oil (88.7%), and nuts (83.4%), as well as lower consumption of carbonated beverages (96.4%), red meat (96.0%), and butter or margarine (97.2%).

Additional cluster analyses stratified by sex and age were performed to explore differences in Mediterranean dietary patterns across these subgroups (Figures S1–S7). The MEDAS item with the greatest weight in cluster differentiation was vegetable consumption among women and fruit consumption among men. In the age-stratified analyses, the most influential items were vegetable consumption at age 25 and 35, sugary or carbonated beverage consumption at age 45, pulse consumption at age 55, and fruit consumption at age 65.

Table 4 shows that Cluster 2 had a significantly lower mean PSQI score than Cluster 1 (6.6 vs. 7.5; *p* = 0.021), indicating better subjective sleep quality. In contrast, no significant differences were observed between clusters for sleep efficiency (*p* = 0.544), WASO (*p* = 0.611), number of awakenings (*p* = 0.405), or fragmentation index (*p* = 0.125). These analyses were adjusted for age, sex, educational level, employment status, CCI, BMI, caffeine and alcohol consumption, smoking status, and physical activity.

## 4. Discussion

The results of this study in 500 Spanish adults confirm that greater adherence to the MD is associated with better self-reported sleep quality (measured using the PSQI), without corresponding changes in objective actigraphy parameters. Specifically, cluster analysis identified that a dietary profile characterized by the consumption of 3 or more servings of fruit and 2 or more servings of vegetables per day is associated with a higher perception of sleep quality.

This finding is particularly relevant in a general, non-clinical population setting, as it suggests that the benefits of the Mediterranean dietary pattern on sleep quality are not limited to populations with pre-existing conditions but may also be observed in the general population, supporting its role as a potential health promotion strategy.

### 4.1. Association Between the Mediterranean Diet and Subjective Sleep Quality

Our results, showing a 0.187-point reduction in the PSQI for every one-point increase in the MEDAS score, are consistent with the current literature linking diet quality to sleep perception. Specifically, Godos et al. [35] concluded that individuals with higher adherence to the MD are 10% more likely to report adequate sleep quality for each one-point increase in the adherence score. Furthermore, when adherence was analyzed categorically, high MD adherence was associated with lower odds of poor sleep quality; however, this association did not reach statistical significance after adjustment for the additional confounders (OR = 0.685; 95% CI: 0.454–1.034; *p* = 0.072). It should be noted that, although the absolute magnitude of the effect on the PSQI may seem modest, such reductions are comparable to those observed in non-pharmacological behavioral interventions aimed at improving sleep quality in the general population, underscoring the clinical relevance of dietary patterns as a modifiable factor [36].

Of particular relevance to our geographical context is the study by Ramón-Arbués et al. [37] in a Spanish university population. Their findings were even more pronounced than ours, reporting that individuals with low-quality diets were four times more likely to experience poor sleep quality (OR = 4.20; 95% CI: 2.07 to 8.52). Furthermore, when examining food groups separately, they identified insufficient intake of fruits (OR = 4.08) and vegetables (OR = 1.63) as the strongest dietary predictors of poor sleep. This is consistent with our cluster analysis results, in which Cluster 2, characterized by higher consumption of fruits and vegetables, showed significantly better PSQI scores.

Possible biological mechanisms suggest that the MD, being rich in antioxidants (polyphenols), unsaturated fatty acids, fiber, and vitamins, may influence sleep through three synergistic pathways. First, biochemical regulation: key nutrients such as tryptophan (abundant in fish, legumes, and nuts, with higher consumption in Cluster 2 of our study) are direct precursors of serotonin and melatonin, hormones that modulate the sleep–wake cycle [38]. The concomitant intake of complex carbohydrates facilitates the transport of tryptophan to the brain by reducing competition with other neutral amino acids [39]. This effect may not necessarily translate into changes in sleep duration or efficiency, but rather into improved perception of sleep depth and morning recovery, dimensions captured by the PSQI. Second, inflammation and oxidative stress may also play a role: the MD is consistently associated with lower levels of inflammatory markers (such as IL-6 and TNF- α). Since inflammation is implicated in sleep disruption and daytime fatigue, the anti-inflammatory effects of polyphenols and omega-3 fatty acids may contribute to improved sleep stability and perceived restfulness [39]. Finally, the gut–brain axis should be considered: high intake of prebiotic fiber modulates gut microbiome composition, promoting the production of metabolites (such as butyrate) and neurotransmitters that signal to the central nervous system [40]. This pathway could explain improvements in stress regulation, nocturnal autonomic tone, and overall perception of rest, even in the absence of detectable changes in actigraphic parameters [41,42].

### 4.2. The Discrepancy Between Subjective and Objective Sleep

A finding consistent with previous research is the difference in the association between the MD and the type of sleep measurement. Our data show a significant relationship between the MEDAS score and subjective sleep quality measured by the PSQI, whereas objective parameters derived from actigraphy were not associated with dietary adherence. This pattern has been described previously, with some studies reporting clearer effects of health dietary patterns on perceived sleep quality than on objective physiological parameters [25].

Experimental studies have shown that dietary composition may modify sleep architecture (e.g., by increasing slow-wave sleep or REM sleep), changes that are difficult to detect using actigraphy, as this method primarily relies on body movements [43]. It is possible that participants with high adherence to the MD experience more restorative sleep at the neurochemical level, which improves their morning satisfaction and PSQI scores, without necessarily altering nocturnal motor immobility [44].

Furthermore, Rezaie et al. [45] note that psychological factors such as stress and mood may act as mediators in this discrepancy between perceived and objectively measured sleep. Given that the MD has been consistently associated with lower levels of depression and anxiety, it is possible that the association observed with the PSQI is mediated by improvements in general psychological well-being, leading individuals to perceive their sleep more positively [46].

### 4.3. Strengths and Limitations

Among the strengths of this study are the large and representative sample size, the use of both objective (actigraphy) and subjective (PSQI) sleep assessment methods, and adjustment for multiple relevant confounding factors. However, the cross-sectional nature of the study limits causal inference; it is not possible to determine whether greater adherence to the MD promotes better sleep or whether individuals with better sleep patterns are more likely to adopt healthier dietary habits. Furthermore, although actigraphy is more objective than questionnaire-based assessments, it lacks the precision of polysomnography in distinguishing sleep stages, which may mask potential effects of diet on slow-wave or REM sleep. Additionally, although the actigraphy protocol included at least one weekend day, the five-day monitoring period may not have fully captured weekly variability in sleep patterns. Another limitation is the reliance on the MEDAS as a measure of dietary quality, which, although validated, does not quantify the exact intake of specific nutrients that may mediate the relationship with sleep (e.g., tryptophan or polyphenols).

## 5. Conclusions

In conclusion, our results show that higher MEDAS scores were independently associated with improved subjective sleep quality, as assessed by the PSQI, while no associations were observed with objective sleep parameters measured by actigraphy. These findings suggest that the MD may primarily influence the perception of nighttime rest rather than its objective physiological characteristics, at least in general populations without diagnosed sleep disorders. Although the cross-sectional design limits causal inference, these findings highlight the potential role of MD as a modifiable lifestyle factor related to well-being and sleep quality, and underscore the need for longitudinal studies and controlled interventions to clarify the underlying mechanisms and the directionality of these associations.

## Figures and Tables

**Figure 1:**
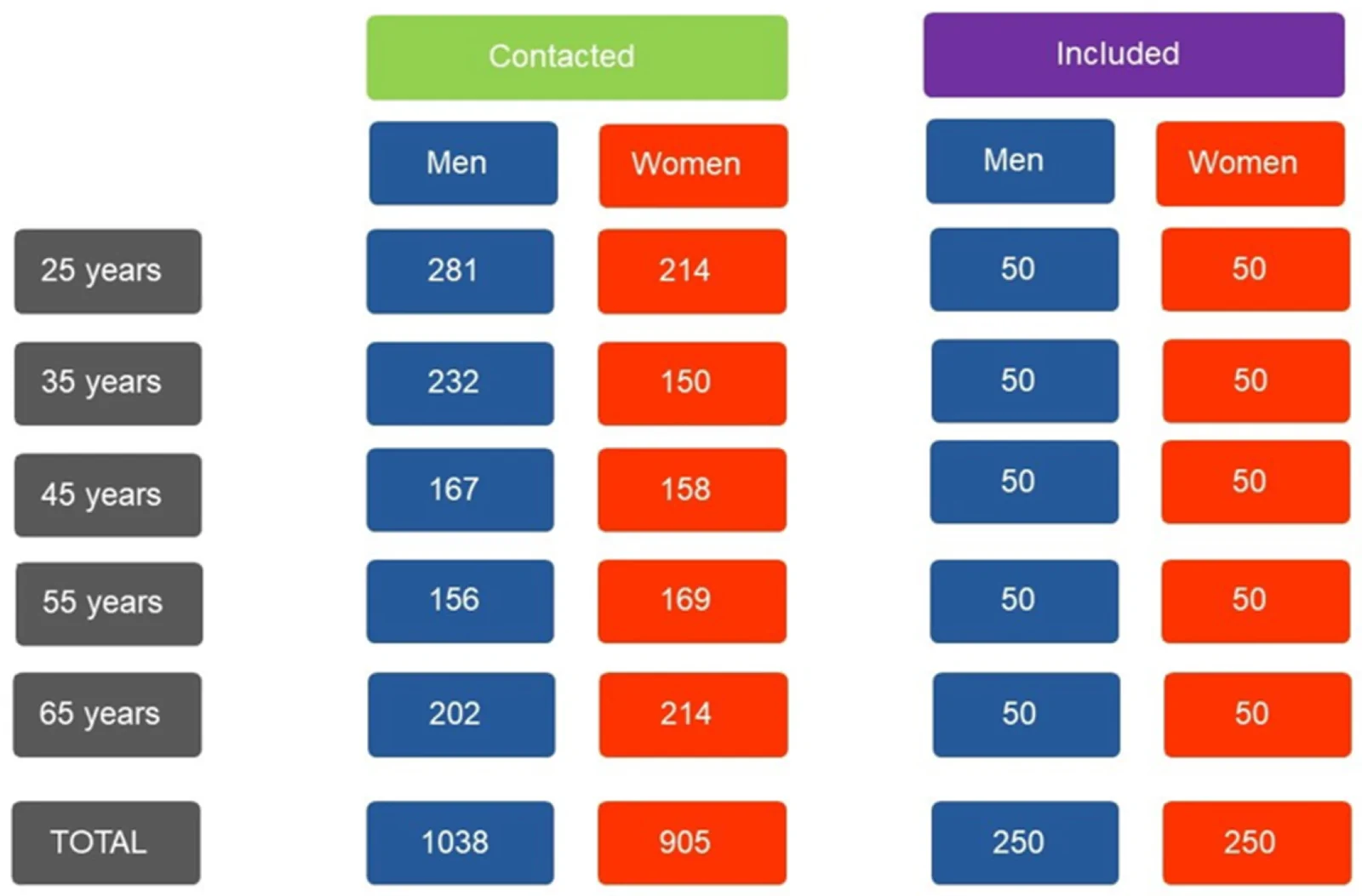
Individuals contacted and included in the study, stratified by age group and sex.

**Figure 2:**
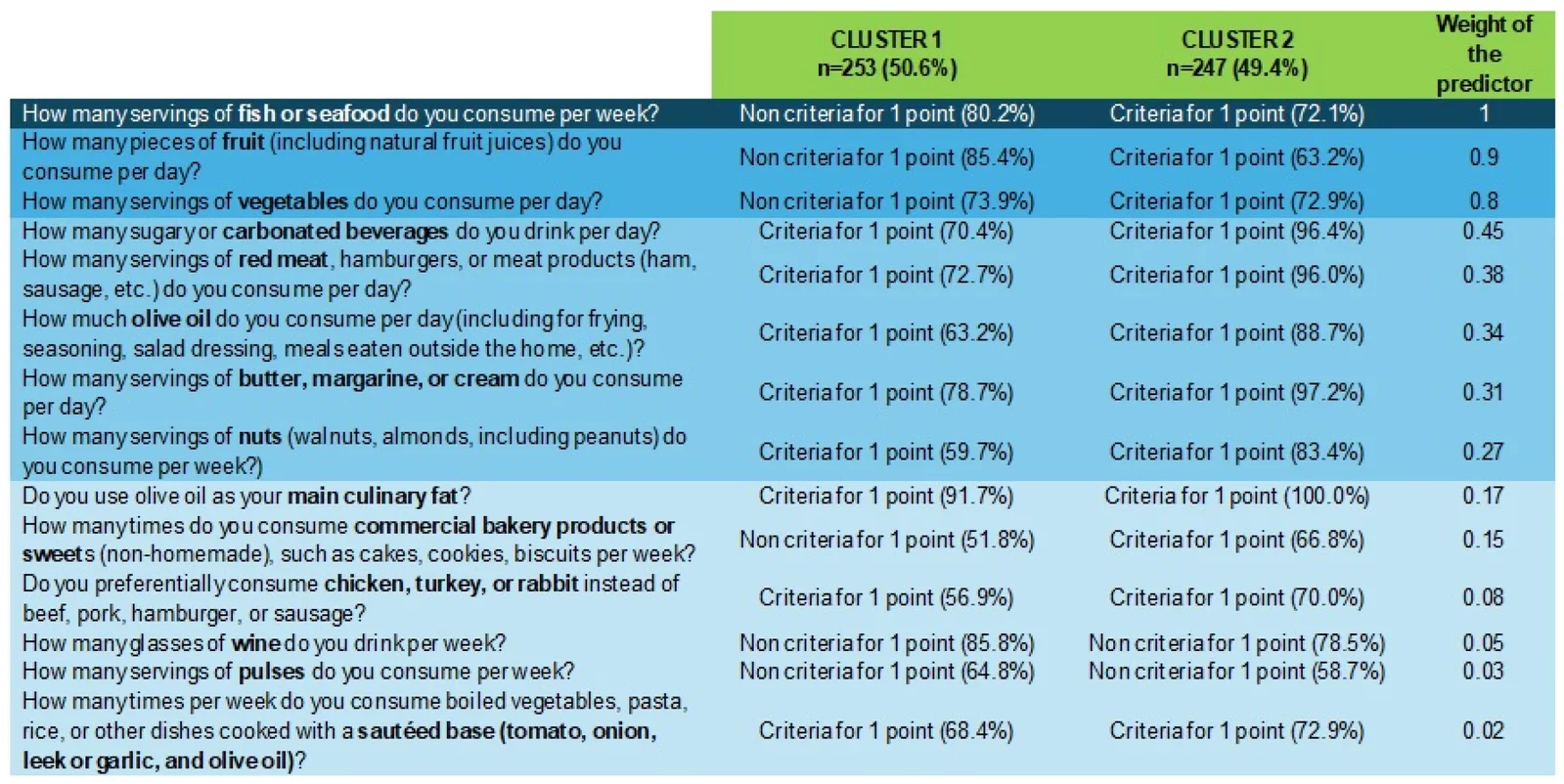
Characterization of the clusters based on adherence to each MEDAS item.

**Table 1:** Clinical and sociodemographic characteristics.

	Overall (*n* = 500)
Marital Status, n (%)	
Married/Unmarried Union	276 (55.2)
Separated/Divorced	29 (5.8)
Single	185 (37.0)
Others	10 (2.0)
Education level, n (%)	
Elementary school	53 (10.6)
Middle or high school	203 (40.6)
University studies	244 (48.8)
Work situation, n (%)	
Student	8 (1.6)
Employed	328 (65.6)
Maternity leave	3 (0.6)
Unemployed	55 (11.0)
Disability to work	11 (2.2)
Retired	87 (17.4)
Home and childcare	6 (1.2)
Others	2 (0.4)
Charlson Comorbidity Index, mean ± SD	0.41 ± 0.88

**Table 2:** Mediterranean Diet Adherence Screener components.

	Criteria f1.	Overall (n = 500) N (%)
1. Do you use olive oil as your main culinary fat?	Yes	479 (95.8%)
2. How much olive oil do you consume per day (including frying, seasoning, salad dressing, meals eaten outside the home, etc.)?	≥4 Tablespoons	379 (75.8%)
3. How many servings of vegetables do you consume per day?	≥2 servings	246 (49.2%)
4. How many pieces of fruit (including natural fruit juices) do you consume per day?	≥3 pieces	193 (38.6%)
5. How many servings of red meat, hamburgers, or meat products (ham, sausage, etc.) do you consume per day?	<1 servings	421 (84.2%)
6. How many servings of butter, margarine, or cream do you consume per day?	<1 servings	439 (87.8%)
7. How many sugary or carbonated beverages do you drink per day?	<1 beverages	416 (83.2%)
8. How many glasses of wine do you drink per week?	≥7 glasses	89 (17.8%)
9. How many servings of pulses do you consume per week?	≥3 servings	191 (38.2%)
10. How many servings of fish or seafood do you consume per week?	≥3 servings	228 (45.6%)
11. How many times do you consume commercial bakery products or sweets (non-homemade), such as cakes, cookies, biscuits per week?	<3 servings	287 (57.4%)
12. How many servings of nuts (walnuts, almonds, including peanuts) do you consume per week?)	≥3 servings	357 (71.4%)
13. Do you preferentially consume chicken, turkey, or rabbit instead of beef, pork, hamburger, or sausage?	Yes	317 (63.4%)
14. How many times per week do you consume boiled vegetables, pasta, rice, or other dishes cooked with a sautéed base (tomato, onion, leek or garlic, and olive oil)?	≥2 servings	353 (70.6%)
High adherence to the Mediterranean Diet (Score ≥ 9)		286 (57.2%)
MEDAS score		8.8 ± 2.2
MEDAS: Mediterranean Diet Adherence Screener.		

**Table 3:** Multiple regressions with different sleep quality parameters as dependent variables and Mediterranean diet adherence score as independent variable.

Dependent Variable	Independent Variable	Unstandardized Coefficient β and 95% Confidence Interval	t	*p* Value
PSQI score	MEDAS score	−0.187 (−0.354 to −0.021)	−2.215	0.027
Efficiency	MEDAS score	−0.053 (−0.217 to 0.111)	−0.634	0.526
WASO	MEDAS score	0.119 (−0.613 to 0.850)	0.319	0.750
Number of Awakenings	MEDAS score	0.122 (−0.103 to 0.347)	1.067	0.286
Sleep Fragmentation Index	MEDAS score	0.278 (−0.030 to 0.586)	1.775	0.076

All multiple regressions included the main determinants of sleep quality (age, sex, level of education, employment status, Charlson comorbidity index, BMI, daily caffeine, alcohol consumption, smoking status and physical activity levels) as independent variables. Data relating to these independent variables does not appear in this table. PSQI: Pittsburgh Sleep Quality Index; WASO: Wake After Sleep Onset; MEDAS: Mediterranean Diet Adherence Screener.

**Table 4:** Estimated marginal means of sleep quality indices according to the classification of mediterranean diet clusters.

	Cluster 1 (n = 253)	Cluster 2 (n = 247)		Overall (n = 500)
	Mean (95% CI)	Mean (95% CI)	*p*-value	Mean (SD)
PSQI score	7.5 (7.0–8.0)	6.6 (6.1–7.1)	0.021	7.1 (4.1)
Efficiency	90.9 (90.5–91.4)	90.7 (90.2–91.2)	0.544	90.8 (4.2)
WASO	36.9 (34.7–39.1)	37.8 (35.5–40.0)	0.611	37.6 (18.3)
Number of Awakenings	13.7 (13.0–14.4)	14.1 (13.4–14.8)	0.405	13.9 (5.6)
Sleep Fragmentation Index	23.4 (22.5–24.3)	24.5 (23.5–25.4)	0.125	23.9 (7.5)

Estimated marginal means adjusted for age, sex, level of education, employment status, Charlson comorbidity index, BMI, caffeine and alcohol consumption, smoking status, and physical activity. PSQI: Pittsburgh Sleep Quality Index; WASO: Wake After Sleep Onset; CI: Confidence Interval; SD: Standard Deviation.

## Data Availability

The raw data supporting the conclusions of this article will be made available by the authors on request.

## Supplementary Materials

The following supporting information can be downloaded at https://www.mdpi.com/article/10.3390/nu18203305/s1: Table S1. STROBE; Figure S1. Characterization of the clusters based on adherence to each MEDAS item, stratified by sex: women. Figure S2. Characterization of the clusters based on adherence to each MEDAS item, stratified by sex: men. Figure S3. Characterization of the clusters based on adherence to each MEDAS item, stratified by age: 25 years. Figure S4. Characterization of the clusters based on adherence to each MEDAS item, stratified by age: 35 years. Figure S5. Characterization of the clusters based on adherence to each MEDAS item, stratified by age: 45 years. Figure S6. Characterization of the clusters based on adherence to each MEDAS item, stratified by age: 55 years. Figure S7. Characterization of the clusters based on adherence to each MEDAS item, stratified by age: 65 years.

## Abbreviations

The following abbreviations are used in this manuscript:

PSQI	Pittsburgh Sleep Quality Index
MD	Mediterranean Diet
SACYL	Servicio de Salud de Castilla y León
CCI	Charlson Comorbidity Index
BMI	Body mass index
ANCOVA	Analysis of Covariance
MEDAS	Mediterranean Diet Adherence Screener
