# Supplementary material for: Mediterranean Diet Adherence and Sleep Quality in Spanish Adults: Divergent Associations Between Subjective and Objective Measures
Source: Nutrients. 2026 Oct 9;18(20):3305. doi: 10.3390/nu18203305 (PMC13708225; doi:10.3390/nu18203305)
Supplement: Supplementary file 1 [file nutrients-18-03305-s001.zip › nutrients-4592052-supplementary.pdf]

Table S1. STROBE

Lachat C et al. (2016) STrengthening the Reporting of OBservational studies in Epidemiology

Item	Item nr	STROBE recommendations	Reported on page #
**Title and abstract**	1	(a) Indicate the study's design with a commonly used term in the title or the abstract.	**Page 1 Lines 1-36**
		(b) Provide in the abstract an informative and balanced summary of what was done and what was found.	**Page 1 Lines 1-36**
**Introduction**			
Background rationale	2	Explain the scientific background and rationale for the investigation being reported.	**Page 1-3 Lines 39-99**
Objectives	3	State specific objectives, including any pre-specified hypotheses.	**Page 3 Lines 105-108**
**Methods**			
Study design	4	Present key elements of study design early in the paper.	**Page 3 Lines 110-114**

Item	Item nr	STROBE recommendations	Reported on page #
Settings	5	Describe the setting, locations, and relevant dates, including periods of recruitment, exposure, follow-up, and data collection.	**Page 3 Lines 116-122**
Participants	6	a) Cohort study—Give the eligibility criteria, and the sources and methods of selection of participants. Describe methods of follow-up.<br><br>Case-control study—Give the eligibility criteria, and the sources and methods of case ascertainment and control selection. Give the rationale for the choice of cases and controls.<br><br>Cross-sectional study—Give the eligibility criteria, and the sources and methods of selection of participants.<br><br>(b) Cohort study—For matched studies, give matching criteria and number of exposed and unexposed.<br><br>Case-control study—For matched studies, give matching criteria and the number of controls per case.	**Pages 4**<br><br>**Lines 127-133**
Variables	7	Clearly define all outcomes, exposures, predictors, potential confounders, and effect modifiers. Give diagnostic criteria, if applicable.	**Pages 4-5 134-186**

Item	Item nr	STROBE recommendations	Reported on page #
Data sources - measurements	8	For each variable of interest, give sources of data and details of methods of assessment (measurement).Describe comparability of assessment methods if there is more than one group.	**Page 4-10 Lines 135-276**
Bias	9	Describe any efforts to address potential sources of bias.	**Page 11 Lines 348-361**
Study Size	10	Explain how the study size was arrived at.	**Page 5 Lines 187 - 192**
Quantitative variables	11	Explain how quantitative variables were handled in the analyses. If applicable, describe which groupings were chosen and why.	
Statistical Methods	12	(a) Describe all statistical methods, including those used to control for confounding<br>(b) Describe any methods used to examine subgroups and interactions.<br>(c) Explain how missing data were addressed.<br>(d) Cohort study—If applicable, explain how loss to follow-up was addressed.	**Pages 5 - 6 Lines 193 - 213**

Item	Item nr	STROBE recommendations	Reported on page #
		Case-control study—If applicable, explain how matching of cases and controls was addressed.<br><br>Cross-sectional study—If applicable, describe analytical methods taking account of sampling strategy.<br><br>(e) Describe any sensitivity analyses.	
**Results**			
Participants	13	(a) Report the numbers of individuals at each stage of the study—e.g., numbers potentially eligible, examined for eligibility, confirmed eligible, included in the study, completing follow-up, and analyzed.<br><br>(b) Give reasons for non-participation at each stage.<br><br>(c) Consider use of a flow diagram.	**Table 1**<br><br>**Figure 1**
Descriptive data	14	(a) Give characteristics of study participants (e.g., demographic, clinical, social) and information on exposures and potential confounders<br><br>(b) Indicate the number of participants with missing data for each variable of interest	**Tables 1 and 2**

Item	Item nr	STROBE recommendations	Reported on page #
		(c) Cohort study—Summarize follow-up time (e.g., average and total amount)	
Outcome data	15	Cohort study—Report numbers of outcome events or summary measures over time.<br><br>Case-control study—Report numbers in each exposure category, or summary measures of exposure.<br><br>Cross-sectional study—Report numbers of outcome events or summary measures.	**Tables 3 and 4**
Main results	16	(a) Give unadjusted estimates and, if applicable, confounder-adjusted estimates and their precision (e.g., 95% confidence interval).<br><br>Make clear which confounders were adjusted for and why they were included.<br><br>(b) Report category boundaries when continuous variables were categorized.<br><br>(c) If relevant, consider translating estimates of relative risk into absolute risk for a meaningful time period.	**Table 3**

Item	Item nr	STROBE recommendations	Reported on page #
Other analyses	17	Report other analyses done—e.g., analyses of subgroups and interactions and sensitivity analyses.	**Table 4**
**Discussion**			
Key results	18	Summarize key results with reference to study objectives.	**Pages 10 Lines 278**
Limitation	19	Discuss limitations of the study, taking into account sources of potential bias or imprecision. Discuss both direction and magnitude of any potential bias.	**Page 11 Lines 348 - 361**
Interpretation	20	Give a cautious overall interpretation of results considering objectives, limitations, multiplicity of analyses, results from similar studies, and other relevant evidence.	**Pages 10-11 Lines 288 - 347**
Generalizability	21	Discuss the generalizability (external validity) of the study results.	**Pages 10 - 11 Lines 288 - 347**
**Other information**			
Funding	22	Give the source of funding and the role of the funders for the present study and, if applicable, for the original study on which the present article is based.	**Pag 12 Line 379 - 382**

Item	Item nr	STROBE recommendations	Reported on page #
Ethics			**Pag 12 Line 383 - 386**
Supplementary material			

Figure S1. Characterization of the clusters based on adherence to each MEDAS item, stratified by sex: women

	CLUSTER 1<br>n = 158 (63.5 %)	CLUSTER 2<br>n=91 (36.5 %)	Weight of the predictor
How many servings of **vegetables** do you consume per day?	Criteria for 1 point (86.1%)	Non criteria for 1 point (78.0%)	1
How many servings of **fish or seafood** do you consume per week?	Criteria for 1 point (72.2%)	Non criteria for 1 point (84.6%)	0.74
How many pieces of **fruit** (including natural fruit juices) do you consume per day?	Criteria for 1 point (57.0%)	Non criteria for 1 point (84.6%)	0.43
How many servings of **butter, margarine, or cream** do you consume per day?	Criteria for 1 point (96.8%)	Criteria for 1 point (71.4%)	0.36
How many sugary or **carbonated beverages** do you drink per day?	Criteria for 1 point (93.7%)	Criteria for 1 point (65.9%)	0.34
How many servings of **red meat**, hamburgers, or meat products (ham, sausage, etc.) do you consume per day?	Criteria for 1 point (95.6%)	Criteria for 1 point (71.4%)	0.31
How many servings of **nuts** (walnuts, almonds, including peanuts) do you consume per week?)	Criteria for 1 point (80.4%)	Criteria for 1 point (56.0%)	0.19
How many times do you consume **commercial bakery products or sweet**s (non-homemade), such as cakes, cookies, biscuits per week?	Criteria for 1 point (75.3%)	Criteria for 1 point (50.5%)	0.18
Do you use olive oil as your **main culinary fat**?	Criteria for 1 point (100%)	Criteria for 1 point (91.2%)	0.17
Do you preferentially consume **chicken, turkey, or rabbit** instead of beef, pork, hamburger, or sausage?	Criteria for 1 point (77.8%)	Criteria for 1 point (57.1%)	0.14
How much **olive oil** do you consume per day (including for frying, seasoning, salad dressing, meals eaten outside the home, etc.)?	Criteria for 1 point (84.2%)	Criteria for 1 point (70.3%)	0.09
How many times per week do you consume boiled vegetables, pasta, rice, or other dishes cooked with a **sautéed base (tomato, onion, leek or garlic, and olive oil)**?	Criteria for 1 point (75.9%)	Criteria for 1 point (60.4%)	0.09
How many servings of **pulses** do you consume per week?	Non criteria for 1 point (62.0%)	Non criteria for 1 point (75.8%)	0.07
How many glasses of **wine** do you drink per week?	Non criteria for 1 point (86.1%)	Non criteria for 1 point (89.0%)	0.01

Figure S2. Characterization of the clusters based on adherence to each MEDAS item, stratified by sex: men

	CLUSTER 1 n = 121 (48.4 %)	CLUSTER 2 n=129 (51.6 %)	Weight of the predictor
How many pieces of **fruit** (including natural fruit juices) do you consume per day?	Criteria for 1 point (58.7%)	Non criteria for 1 point (80.6%)	1
How many servings of **red meat**, hamburgers, or meat products (ham, sausage, etc.) do you consume per day?	Criteria for 1 point (97.5%)	Criteria for 1 point (66.7%)	0.74
How many sugary or **carbonated beverages** do you drink per day?	Criteria for 1 point (97.5%)	Criteria for 1 point (69.8%)	0.65
How many servings of **vegetables** do you consume per day?	Criteria for 1 point (52.9%)	Non criteria for 1 point (80.6%)	0.59
How many servings of **fish or seafood** do you consume per week?	Criteria for 1 point (57.0%)	Non criteria for 1 point (76.0%)	0.55
How many servings of **nuts** (walnuts, almonds, including peanuts) do you consume per week?)	Criteria for 1 point (85.1%)	Criteria for 1 point (58.1%)	0.44
How many glasses of **wine** do you drink per week?	Non criteria for 1 point (64.5%)	Non criteria for 1 point (89.1%)	0.43
How much **olive oil** do you consume per day (including for frying, seasoning, salad dressing, meals eaten outside the home, etc.)?	Criteria for 1 point (85.1%)	Criteria for 1 point (60.5%)	0.38
How many times do you consume **commercial bakery products or sweet**s (non-homemade), such as cakes, cookies, biscuits per week?	Criteria for 1 point (61.2%)	Non criteria for 1 point (63.6%)	0.32
Do you use olive oil as your **main culinary fat**?	Criteria for 1 point (100%)	Criteria for 1 point (89.9%)	0.27
How many servings of **butter, margarine, or cream** do you consume per day?	Criteria for 1 point (93.4%)	Criteria for 1 point (83.7%)	0.14
How many servings of **pulses** do you consume per week?	Non criteria for 1 point (51.2%)	Non criteria for 1 point (61.2%)	0.07
Do you preferentially consume **chicken, turkey, or rabbit** instead of beef, pork, hamburger, or sausage?	Criteria for 1 point (55.4%)	Criteria for 1 point (57.4%)	0.01
How many times per week do you consume boiled vegetables, pasta, rice, or other dishes cooked with a **sautéed base (tomato, onion, leek or garlic, and olive oil)**?	Criteria for 1 point (71.9%)	Criteria for 1 point (70.5%)	0.01

Figure S3. Characterization of the clusters based on adherence to each MEDAS item, stratified by age: 25 years.

	CLUSTER 1<br>n = 59 (59 %)	CLUSTER 2<br>n=41 (41 %)	Weight of the predictor
How many servings of **vegetables** do you consume per day?	Criteria for 1 point (74.6%)	Non criteria for 1 point (87.8%)	1
How many pieces of **fruit** (including natural fruit juices) do you consume per day?	Criteria for 1 point (50.8%)	Non criteria for 1 point (97.6%)	0.72
How many servings of **fish or seafood** do you consume per week?	Non criteria for 1 point (54.2%)	Non criteria for 1 point (100%)	0.71
How many times do you consume **commercial bakery products or sweet**s (non-homemade), such as cakes, cookies, biscuits per week?	Criteria for 1 point (72.9%)	Non criteria for 1 point (65.9%)	0.43
How many sugary or **carbonated beverages** do you drink per day?	Criteria for 1 point (93.2%)	Criteria for 1 point (65.9%)	0.37
How many servings of **nuts** (walnuts, almonds, including peanuts) do you consume per week?)	Criteria for 1 point (84.7%)	Criteria for 1 point (53.7%)	0.35
Do you use olive oil as your **main culinary fat**?	Criteria for 1 point (100%)	Criteria for 1 point (90.2%)	0.20
How many times per week do you consume boiled vegetables, pasta, rice, or other dishes cooked with a **sautéed base (tomato, onion, leek or garlic, and olive oil)**?	Criteria for 1 point (88.1%)	Criteria for 1 point (68.3%)	0.20
How much **olive oil** do you consume per day (including for frying, seasoning, salad dressing, meals eaten outside the home, etc.)?	Criteria for 1 point (72.9%)	Criteria for 1 point (51.2%)	0.17
How many servings of **butter, margarine, or cream** do you consume per day?	Criteria for 1 point (91.5%)	Criteria for 1 point (82.9%)	0.08
How many glasses of **wine** do you drink per week?	Non criteria for 1 point (91.5%)	Non criteria for 1 point (82.9%)	0.08
How many servings of **red meat**, hamburgers, or meat products (ham, sausage, etc.) do you consume per day?	Criteria for 1 point (76.3%)	Criteria for 1 point (70.7%)	0.03
How many servings of **pulses** do you consume per week?	Non criteria for 1 point (57.6%)	Non criteria for 1 point (58.5%)	0.00
Do you preferentially consume **chicken, turkey, or rabbit** instead of beef, pork, hamburger, or sausage?	Criteria for 1 point (71.2%)	Criteria for 1 point (70.7%)	0.00

Figure S4. Characterization of the clusters based on adherence to each MEDAS item, stratified by age: 35 years.

	CLUSTER 1 n=42 (42 %)	CLUSTER 2 n = 58 (58 %)	Weight of the predictor
How many servings of **vegetables** do you consume per day?	Non criteria for 1 point (100%)	Criteria for 1 point (77.6%)	1
How many servings of **nuts** (walnuts, almonds, including peanuts) do you consume per week?)	Non criteria for 1 point (61.9%)	Criteria for 1 point (89.7%)	0.53
How many servings of **fish or seafood** do you consume per week?	Non criteria for 1 point (92.9%)	Criteria for 1 point (53.4%)	0.42
How many servings of **red meat**, hamburgers, or meat products (ham, sausage, etc.) do you consume per day?	Criteria for 1 point (64.3%)	Criteria for 1 point (94.8%)	0.29
How many pieces of **fruit** (including natural fruit juices) do you consume per day?	Non criteria for 1 point (88.1%)	Non criteria for 1 point (53.4%)	0.26
How many times do you consume **commercial bakery products or sweet**s (non-homemade), such as cakes, cookies, biscuits per week?	Non criteria for 1 point (54.8%)	Criteria for 1 point (77.6%)	0.22
Do you preferentially consume **chicken, turkey, or rabbit** instead of beef, pork, hamburger, or sausage?	Non criteria for 1 point (54.8%)	Criteria for 1 point (74.1%)	0.18
How many servings of **pulses** do you consume per week?	Non criteria for 1 point (73.8%)	Non criteria for 1 point (51.7%)	0.12
How much **olive oil** do you consume per day (including for frying, seasoning, salad dressing, meals eaten outside the home, etc.)?	Criteria for 1 point (73.8%)	Criteria for 1 point (89.7%)	0.10
How many glasses of **wine** do you drink per week?	Non criteria for 1 point (97.6%)	Non criteria for 1 point (86.2%)	0.09
Do you use olive oil as your **main culinary fat**?	Criteria for 1 point (92.9%)	Criteria for 1 point (98.3%)	0.06
How many sugary or **carbonated beverages** do you drink per day?	Criteria for 1 point (86.2%)	Criteria for 1 point (76.2%)	0.05
How many servings of **butter, margarine, or cream** do you consume per day?	Criteria for 1 point (90.5%)	Criteria for 1 point (94.8%)	0.03
How many times per week do you consume boiled vegetables, pasta, rice, or other dishes cooked with a **sautéed base (tomato, onion, leek or garlic, and olive oil)**?	Criteria for 1 point (59.5%)	Criteria for 1 point (65.5%)	0.02

Figure S5. Characterization of the clusters based on adherence to each MEDAS item, stratified by age: 45 years.

	CLUSTER 1 n = 52 (52 %)	CLUSTER 2 n=48 (48 %)	Weight of the predictor
How many sugary or **carbonated beverages** do you drink per day?	Criteria for 1 point (61.3%)	Criteria for 1 point (100%)	1
How many pieces of **fruit** (including natural fruit juices) do you consume per day?	Non criteria for 1 point (85.5%)	Criteria for 1 point (55.3%)	0.97
How many servings of **red meat**, hamburgers, or meat products (ham, sausage, etc.) do you consume per day?	Criteria for 1 point (66.1%)	Criteria for 1 point (100%)	0.86
How many times do you consume **commercial bakery products or sweet**s (non-homemade), such as cakes, cookies, biscuits per week?	Non criteria for 1 point (56.5%)	Criteria for 1 point (84.2%)	0.85
How many servings of **vegetables** do you consume per day?	Non criteria for 1 point (51.6%)	Criteria for 1 point (81.6%)	0.61
How many servings of **butter, margarine, or cream** do you consume per day?	Criteria for 1 point (72.6%)	Criteria for 1 point (97.4%)	0.56
How many glasses of **wine** do you drink per week?	Non criteria for 1 point (79.0%)	Non criteria for 1 point (100%)	0.52
How much **olive oil** do you consume per day (including for frying, seasoning, salad dressing, meals eaten outside the home, etc.)?	Criteria for 1 point (71.0%)	Criteria for 1 point (94.7%)	0.48
How many servings of **nuts** (walnuts, almonds, including peanuts) do you consume per week?)	Criteria for 1 point (67.7%)	Criteria for 1 point (86.8%)	0.30
Do you preferentially consume **chicken, turkey, or rabbit** instead of beef, pork, hamburger, or sausage?	Criteria for 1 point (59.7%)	Criteria for 1 point (78.9%)	0.27
Do you use olive oil as your **main culinary fat**?	Criteria for 1 point (93.5%)	Criteria for 1 point (100%)	0.19
How many servings of **fish or seafood** do you consume per week?	Criteria for 1 point (51.6%)	Non criteria for 1 point (60.5%)	0.13
How many servings of **pulses** do you consume per week?	Non criteria for 1 point (61.3%)	Non criteria for 1 point (50.0%)	0.12
How many times per week do you consume boiled vegetables, pasta, rice, or other dishes cooked with a **sautéed base (tomato, onion, leek or garlic, and olive oil)**?	Criteria for 1 point (53.2%)	Criteria for 1 point (60.5%)	0.07

Figure S6. Characterization of the clusters based on adherence to each MEDAS item, stratified by age: 55 years.

	**CLUSTER 1 n = 60 (60 %)**	**CLUSTER 2 n=40 (40 %)**	**Weight of the predictor**
How many servings of **pulses** do you consume per week?	Non criteria for 1 point (78.3%)	Criteria for 1 point (72.5%)	1
How many servings of **nuts** (walnuts, almonds, including peanuts) do you consume per week?)	Criteria for 1 point (56.7%)	Criteria for 1 point (100%)	0.93
How many pieces of **fruit** (including natural fruit juices) do you consume per day?	Non criteria for 1 point (70.0%)	Criteria for 1 point (72.5%)	0.71
How many servings of **red meat**, hamburgers, or meat products (ham, sausage, etc.) do you consume per day?	Criteria for 1 point (98.3%)	Criteria for 1 point (82.5%)	0.37
How many servings of **vegetables** do you consume per day?	Non criteria for 1 point (71.7%)	Criteria for 1 point (55.0%)	0.34
Do you preferentially consume **chicken, turkey, or rabbit** instead of beef, pork, hamburger, or sausage?	Criteria for 1 point (65.0%)	Non criteria for 1 point (60.0%)	0.29
How many times do you consume **commercial bakery products or sweet**s (non-homemade), such as cakes, cookies, biscuits per week?	Criteria for 1 point (60.0%)	Non criteria for 1 point (65.0%)	0.29
How many times per week do you consume boiled vegetables, pasta, rice, or other dishes cooked with a **sautéed base (tomato, onion, leek or garlic, and olive oil)**?	Criteria for 1 point (85.0%)	Criteria for 1 point (97.5%)	0.22
How much **olive oil** do you consume per day (including for frying, seasoning, salad dressing, meals eaten outside the home, etc.)?	Criteria for 1 point (73.3%)	Criteria for 1 point (85.0%)	0.12
Do you use olive oil as your **main culinary fat**?	Criteria for 1 point (96.7%)	Criteria for 1 point (100%)	0.10
How many servings of **fish or seafood** do you consume per week?	Non criteria for 1 point (50.0%)	Criteria for 1 point (60.0%)	0.08
How many servings of **butter, margarine, or cream** do you consume per day?	Criteria for 1 point (90.0%)	Criteria for 1 point (85.0%)	0.05
How many glasses of **wine** do you drink per week?	Non criteria for 1 point (83.3%)	Non criteria for 1 point (77.5%)	0.05
How many sugary or **carbonated beverages** do you drink per day?	Criteria for 1 point (90.0%)	Criteria for 1 point (90.0%)	0.00

Figure S7. Characterization of the clusters based on adherence to each MEDAS item, stratified by age: 65 years.

	CLUSTER 1 n = 54 (54 %)	CLUSTER 2 n=46 (46 %)	Weight of the predictor
How many pieces of **fruit** (including natural fruit juices) do you consume per day?	Criteria for 1 point (85.2%)	Non criteria for 1 point (84.8%)	1
How many times per week do you consume boiled vegetables, pasta, rice, or other dishes cooked with a **sautéed base (tomato, onion, leek or garlic, and olive oil)**?	Criteria for 1 point (87.0%)	Non criteria for 1 point (63.0%)	0.58
How much **olive oil** do you consume per day (including for frying, seasoning, salad dressing, meals eaten outside the home, etc.)?	Criteria for 1 point (92.6%)	Criteria for 1 point (52.2%)	0.46
How many servings of **fish or seafood** do you consume per week?	Criteria for 1 point (81.5%)	Non criteria for 1 point (52.2%)	0.29
Do you use olive oil as your **main culinary fat**?	Criteria for 1 point (100%)	Criteria for 1 point (84.8%)	0.22
How many servings of **nuts** (walnuts, almonds, including peanuts) do you consume per week?)	Criteria for 1 point (75.9%)	Criteria for 1 point (58.7%)	0.10
How many servings of **vegetables** do you consume per day?	Criteria for 1 point (59.3%)	Non criteria for 1 point (56.5%)	0.08
How many sugary or **carbonated beverages** do you drink per day?	Criteria for 1 point (90.7%)	Criteria for 1 point (80.4%)	0.07
Do you preferentially consume **chicken, turkey, or rabbit** instead of beef, pork, hamburger, or sausage?	Criteria for 1 point (55.6%)	Non criteria for 1 point (69.6%)	0.07
How many times do you consume **commercial bakery products or sweet**s (non-homemade), such as cakes, cookies, biscuits per week?	Criteria for 1 point (63.0%)	Criteria for 1 point (50.0%)	0.06
How many servings of **pulses** do you consume per week?	Non criteria for 1 point (70.4%)	Non criteria for 1 point (80.4%)	0.05
How many servings of **butter, margarine, or cream** do you consume per day?	Criteria for 1 point (85.2%)	Criteria for 1 point (91.3%)	0.04
How many glasses of **wine** do you drink per week?	Non criteria for 1 point (61.1%)	Non criteria for 1 point (67.4%)	0.02
How many servings of **red meat**, hamburgers, or meat products (ham, sausage, etc.) do you consume per day?	Criteria for 1 point (92.6%)	Criteria for 1 point (95.7%)	0.02
